# Engineering the Biological Identity of Poly(lactic-co-glycolic Acid) Nanocarriers: Protein-Corona Dynamics, Functional Reprogramming, and Translational Fate

**DOI:** 10.3390/cimb48101037

**Published:** 2026-10-06

**Authors:** Hossein Omidian, Nazanin Kianinejad, Luigi X. Cubeddu

**Affiliations:** 1 Department of Pharmaceutical Sciences, Barry and Judy Silverman College of Pharmacy, Nova Southeastern University, Fort Lauderdale, FL 33328, USA; lcubeddu@nova.edu; 2 Gregory School of Pharmacy, Palm Beach Atlantic University, West Palm Beach, FL 33401, USA; kianin@pba.edu

**Keywords:** PLGA nanocarriers, protein corona, biological identity, bio-nano interface, translational nanomedicine

## Abstract

Poly(lactic-co-glycolic acid) (PLGA) nanocarriers can be engineered by varying polymer architecture, particle dimensions, surface chemistry, ligands, coatings, and cargo; however, biological exposure subsequently remodels the interface encountered by cells and tissues. This narrative review examines how this acquired biological identity may help explain translational failure between favorable formulation-level or in vitro properties and downstream in vivo performance. Across experimental and preclinical studies, serum or tissue-fluid composition, competitive protein exchange, physiological flow, complement and immunoglobulin recognition, ligand masking, tissue sequestration, and altered protein accessibility can redirect uptake, clearance, barrier interaction, biodistribution, efficacy, and safety. Corona formation, however, is not uniformly detrimental: selected interfaces can support dendritic-cell activation, blood-cell hitchhiking, or endogenous apolipoprotein-mediated targeting. We organize the literature as a biological stress-test continuum extending from the engineered pre-corona state through the acquired interface, cellular function, tissue transport, and whole-organism fate, while explicitly distinguishing direct PLGA evidence, mechanistically supported PLGA findings, associative PLGA observations, and comparator-only evidence. No clinical data or meta-analysis are included. The central translational implication is that PLGA performance should be judged by the robustness of its intended function after biologically relevant reprogramming rather than by pristine-particle properties alone.

## 1. Introduction

PLGA nanocarriers have been investigated for drug and gene delivery, imaging, immunotherapy, antimicrobial therapy, and tissue-directed applications because polymer architecture, fabrication, dimensions, charge, stabilizer, ligand display, and payload can all be varied within a biodegradable platform [1,2,3,4,5,6,7]. This flexibility has yielded conventional PLGA nanoparticles, PLGA systems incorporating poly(ethylene glycol) (PEG), hybrid particles, protein-coated carriers, multilayer constructs, and biomimetic membrane-coated formulations. The application range continues to expand; for example, a 2026 report described PLGA-coated Prussian blue nanocubes in an inflammatory bowel disease model using real-time photoacoustic imaging [8]. This example illustrates the breadth of current PLGA applications but is not used as evidence for a protein-corona mechanism in the synthesis that follows.

Exposure to serum, plasma, blood, cerebrospinal fluid, vitreous fluid, amniotic fluid, or other complex biological media creates an acquired interface through the adsorption, exchange, and reorganization of proteins and other biomolecules. This remodeling can alter hydrodynamic dimensions, apparent surface charge, stability, immune recognition, endothelial adhesion, tissue transport, and biodistribution [6,7,8,9,10,11]. Human and mouse serum can generate distinct coronas [10,11]; human serum and fetal bovine serum (FBS) can alter corona composition [12] and leukocyte uptake [13]; and anatomically relevant fluids create additional context-specific identities [8,14,15]. Thus, the physicochemical object characterized immediately after synthesis is only the starting state of a PLGA carrier, not necessarily the biological entity subsequently encountered by cells, tissues, and organs.

This distinction matters because translational failure can arise at several interfaces. Similar total adsorption can conceal compositional differences [11], whereas stabilizers can change total protein more than overall composition [16]; serum conditions and analytical workflow can also modify the observed corona [16,17]. Complement recruitment can alter uptake [10,18], immunoglobulins can alter vascular adhesion [9,19], and flow or anticoagulation can change the observed phenotype [14,15]. Serum-enhanced extracellular-matrix binding can restrict tissue transport [20], while favorable in vitro performance can decouple from tumor accumulation [17,18]; biodegradability does not ensure vascular safety. Conversely, the acquired interface can be exploited: C3-enriched PLGA coronas have accompanied dendritic-cell immunotherapy [5], and recruited apolipoproteins can provide endogenous receptor-mediated targeting [19,21]. A simple stealth-versus-opsonization model is therefore insufficient.

This review addresses an interpretive rather than a descriptive gap. PLGA studies often examine formulation, corona composition, cellular uptake, biodistribution, or efficacy as separate endpoints, making it difficult to determine when an engineered function survives biological exposure and when it is redirected. Rather than cataloging coatings or corona proteins in isolation, we organize the evidence as a design-to-outcome biological stress test: engineered pre-corona identity → dynamic acquired interface → physicochemical/functional reprogramming → cellular and tissue interaction → whole-organism fate. This framework is intended to identify where translational decoupling occurs and which measurements are most informative for downstream function.

We use four terms throughout to distinguish the strength of individual claims. Direct PLGA evidence means that the stated endpoint was measured in a PLGA or PLGA-containing system. Mechanistically supported PLGA evidence refers to a PLGA phenotype strengthened by depletion/reconstitution, receptor blockade, controlled surface perturbation, or another causal intervention; the immunoglobulin depletion/reconstitution experiments in vascular-targeted PLGA are a principal example [22]. Associative PLGA evidence describes a correlation between a PLGA corona feature and a biological outcome without a causal perturbation. Comparator-only evidence refers to non-PLGA materials or geometries used to inform a general biointerface principle; these studies are not used to establish PLGA-specific effects. This vocabulary separates mechanistic inference from association and limits the transfer of conclusions across materially different nanocarriers.

This review provides a qualitative synthesis of experimental and preclinical evidence and does not include clinical data or a meta-analysis. The central questions are which biological identity emerges under the intended exposure conditions; how heterogeneous and stable that identity is; which components remain functionally accessible; and whether the resulting interface preserves, suppresses, or redirects the intended PLGA function.

## 2. Engineering the Pre-Corona Identity

Pre-corona identity refers to the deliberately constructed physicochemical and molecular state of a carrier before exposure to biological fluids. It encompasses polymer composition and architecture, fabrication method, surface-active excipients, PEG content and architecture, coatings and targeting ligands, cargo, particle dimensions, polydispersity, zeta potential, and loading efficiency. Maintaining this distinction prevents downstream protein adsorption or biological responses from being interpreted retrospectively as intrinsic material properties.

### 2.1. Polymer Architecture, Fabrication, and Particle Dimensions

Polymer architecture and manufacturing provide an initial level of control over particle properties. Solvent-diffusion poly(D,L-lactide-co-glycolide) (PLG) nanoparticles carrying a luciferase plasmid were approximately 200 nm in diameter and achieved 80–90% encapsulation efficiency [1]. Here, PLG reflects the terminology used in the original study, whereas PLGA is used throughout this review for poly(lactic-co-glycolic acid). Processing method also influenced particle dimensions: microfluidic fabrication of palmitoylated LL37-loaded PLGA produced substantially smaller and more uniform nanoparticles than bulk nanoprecipitation [23], while rosmarinic-acid-loaded PLGA prepared by Phase Inversion Composition nanoemulsion templating yielded particles of approximately 70–100 nm [24]. Architectural diversity extended beyond conventional PLGA nanospheres to include approximately 20 nm PLGA-block-PEG micelles carrying IR-1061 and terminal gadobutrol [2], PLGA/cholesterol hybrid nanoparticles, lecithin-coated PLGA nanoparticles [3], and layer-by-layer constructs [7].

Particle dimensions were also varied deliberately across PLGA formulations. PLGA and PLGA-PEG nanoparticles were prepared at 100 and 200 nm [25], while Tween-80-stabilized PLGA and poly(L-lactide) nanoparticles were examined at nominal diameters of 50 and 100 nm [26]. These studies illustrate that both particle size and surface architecture can influence protein adsorption; however, within the size range evaluated for the 100 and 200 nm PLGA formulations, surface architecture exerted a greater effect on adsorption behavior than particle size [25].

Polydispersity should be considered a biological-interface variable rather than solely a manufacturing quality metric. A broad particle-size distribution spans a range of curvatures and surface areas, so a nominally single formulation may generate a population of related rather than identical coronas. Bulk Bradford assays or liquid chromatography-tandem mass spectrometry (LC-MS/MS) then yield an ensemble average that may obscure subpopulation-specific adsorption. This is an interpretive consequence rather than a directly demonstrated PLGA mechanism, but it is relevant to the late serum-exposure polydispersity index of 0.60 ± 0.01 reported for one PEGylated PLGA system [27] and to the greater uniformity achieved by microfluidic fabrication [23]. Mean diameter should therefore be interpreted together with polydispersity; when heterogeneity is substantial, bulk proteomics should not be assumed to represent a single biological identity.

Matrix composition and fabrication can also alter properties that are not always carried forward into corona analyses. PLGA and ethyl-cellulose particles prepared by nanoemulsion templating showed formulation-dependent nanomechanical behavior, with polymer composition, surface functionalization, and loading each affecting the mechanical profile [28]. PLGA/cholesterol and cholesterol-only particles developed distinct hard- and soft-corona fingerprints. Together, these observations support treating core composition and interfacial mechanics as features of the starting state rather than as background variables [28].

### 2.2. PEGylation, Stabilization, and Surface Passivation

PEGylation was among the most extensively manipulated pre-corona variables. PEG–polyester nanoparticles spanning 160–270 nm incorporated PEG molecular weights from 2000 to 20,000 g/mol and PEG contents from 0.5 to 20 wt% [29]. In another PLGA system, 750- or 5000-Da PEG was grafted by carbodiimide coupling with N-hydroxysuccinimide or N-hydroxysulfosuccinimide activation, with approximately 44.7% decoration reported for the most extensively modified condition [27]. PLGA-PEG content, D-α-tocopheryl polyethylene glycol succinate (TPGS) concentration, block-copolymer composition, and folate-bearing versus non-targeted PEG were also varied [1,4,24]. PEGylation is therefore an architectural variable rather than a binary descriptor. Curvature adds a further constraint: the same nominal PEG molecular weight and grafting density need not produce the same effective brush conformation on particles of different diameter. Comparator-only studies on polystyrene showed size-dependent effects of PEG/albumin surface modification across 30 nm to 2.6 µm particles [30] and size-dependent circulation effects of poloxamer/poly(ethylene oxide) (PEO) surface coverage on 40, 70, and 137 nm particles [31]. These data do not establish an identical relationship in PLGA, but they caution against treating PEG descriptors as geometry-independent when comparing, for example, 50 and 200 nm carriers. A separate comparator-only polyaspartamide system showed that PEG can be incorporated directly into an amphiphilic polymer architecture to form surfactant-free nanoparticles with PEG detectable at the particle surface [32]. This finding supports PEG incorporation as a pre-corona architectural variable without establishing a PLGA-specific effect on protein adsorption.

PLGA–chitosan–PEG microspheres provide an additional direct PLGA example in which PEG and chitosan were incorporated as surface modifiers while maintaining prolonged release of prodigiosin or paclitaxel [33]. This formulation demonstrates pre-corona surface design but, by itself, does not establish that PEG/chitosan reduced corona formation.

Stabilizers and non-PEG surface chemistries further expanded the range of starting interfaces. Casein, poly(vinyl alcohol), and polyethyleneimine generated negatively charged, near-neutral, and positively charged PLGA nanoparticles [34]. Other systems used cholic acid, Pluronic F127, or poly(vinyl alcohol) [20], whereas Pluronic F68 was applied to magnetic PLGA nanoparticles [35]. Carbohydrate modifications included dextran [2,36], glycol chitosan and carboxymethyl chitosan [37], low-molecular-weight chitosan [38], and glucose functionalization [39]. Ionic-liquid strategies included cholinium fatty-acid coatings, sugar-based ionic-liquid (Glyco-IL) coatings, choline carboxylates, and zwitterionic imidazole-based coatings [7,12,13,40].

Several of these modifications altered the baseline interface before biological-fluid exposure. A zwitterionic imidazole-based ionic-liquid coating increased hydrodynamic radius by nearly 20 nm and shifted surface charge toward neutrality [11]. Low-molecular-weight chitosan-coated PLGA nanoparticles containing diiodinated boron-dipyrromethene (I2BODIPY) remained approximately 147 nm [38], whereas glucosamine modification produced glucose-functionalized PLGA of approximately 125 nm, comparable with unmodified particles [39]. Any subsequent change after serum exposure must therefore be interpreted against the actual modified baseline rather than an assumed unmodified PLGA state.

### 2.3. Ligands, Proteins, Biomimetic Interfaces, and Cargo

Targeting and biomimetic interfaces included cholera toxin subunit B (CTB) [41], anti-transferrin-receptor antibody 8D3 [42], antibodies for targeted tamoxifen delivery [36], adsorbed trastuzumab [43], transactivator-of-transcription peptide (TAT) and acidity-responsive peptide systems [23,44], Arg-Gly-Asp (RGD) [45], folate-bearing PEG [7], and an amyloid-β(1–42)-derived peptide designed to recruit exchangeable apolipoproteins [21]. Albumin, including bovine serum albumin (BSA), was incorporated through several strategies [46,47,48], and cisplatin-loaded PLGA nanoparticles were coated with membranes derived from chimeric antigen receptor T cells (CAR-T) directed against human epidermal growth factor receptor 2 (HER2) [6].

Cargo is also part of the pre-corona state because payload identity and loading can influence release behavior. Examples include small-molecule chemotherapeutics, peptides, cytokines, nucleic acids, antiretroviral agents, immunostimulants, photosensitizers, and imaging probes [2,28,36,45,49,50,51,52]. Defining the starting state is therefore necessary before attributing later behavior to the corona itself.

Starting states were highly formulation specific. Brucine-loaded PLGA-PEG particles ranged from 94 ± 3.05 to 253 ± 8.7 nm, with slightly positive zeta potentials and entrapment efficiencies of 37.5 ± 1.8% to 77 ± 1.3% [53]. Interleukin-12 (IL-12) nanospheres showed major formulation-dependent differences in encapsulation efficiency [54]. A factorially optimized PEGylated docetaxel PLGA/TPGS system was approximately 106 nm, with low polydispersity, a negative zeta potential, and high encapsulation efficiency [4]. These values are not directly comparable across studies, but they illustrate how widely the engineered starting state can vary under the broad label of “PLGA nanoparticle”.

Overall, these studies support a probabilistic rather than deterministic view of pre-corona engineering: surface design biases the interface that may subsequently form but does not itself establish the final biological phenotype. Table 1 consolidates quantitative boundary conditions that are particularly informative for interpreting design–performance relationships.

## 3. Protein-Corona Formation, Composition, and Dynamics

### 3.1. Biological-Fluid, Species, and Concentration Dependence

The biological fluid is a major determinant of the acquired PLGA interface. Human and mouse serum generated substantially different corona profiles, including distinct hard-corona compositions [10,11]. Human serum and fetal bovine serum (FBS) also differed in both total protein adsorption and qualitative corona composition, while increasing the human-serum concentration promoted loss of abundant proteins and recruitment of lower-abundance, higher-affinity species [12]. These PLGA observations are consistent with competitive adsorption and a Vroman-type exchange process, in which initially abundant proteins may be displaced as higher-affinity species compete for the nanoparticle surface; this interpretation is used as a mechanistic framework rather than evidence that a canonical Vroman sequence was directly resolved experimentally [12]. Differences between human-serum and FBS conditions were also reflected in leukocyte interactions, with distinct endocytic patterns observed across human leukocyte populations and complement contributing to uptake of non-surface-engineered PLGA nanoparticles [13].

This dependence extends beyond serum. PLGA and PLGA-PEG nanoparticles exposed to cerebrospinal fluid developed enrichment and depletion patterns distinct from serum-derived coronas and showed altered brain-cell association [58]. In amniotic fluid, core polymer and PEGylation status influenced adsorption, stability, cellular association, and fetal-organ distribution [59]. Vitreous fluid or FBS altered the acquired physicochemical characteristics, uptake, and transfection efficiency of PLGA-containing gene-delivery vectors in primary retinal pigment epithelial cells [60]. These findings favor route- and compartment-specific assessment over reliance on a generic serum model.

### 3.2. Composition, Opsonins, Dysopsonins, and Selective Recruitment

PLGA and PLGA-containing systems displayed diverse classes of corona proteins. Albumin, fibrinogen, immunoglobulin G (IgG), immunoglobulin light chains, apolipoprotein A-I, and apolipoprotein E were identified among adsorbed proteins in PEG–polyester systems [29], whereas human-plasma-exposed uncoated PLGA showed prominent signals for apolipoprotein E, vitronectin, histidine-rich glycoprotein, and kininogen-1 [51]. PLGA and PLGA-PEG coronas also differed in broad protein-class distribution in a brain-cell study [52].

The balance between opsonins and dysopsonins varied markedly among systems. Some ionic-liquid-modified PLGA coronas showed dysopsonin enrichment with opsonin depletion [10], whereas another stabilizer-associated comparison showed enrichment of opsonins and depletion of most dysopsonins [16]. Nanoparticles carrying opsonin-rich hard coronas were rapidly recognized by THP-1 monocytes and macrophage-like cells [61]. In a mechanistic comparator, complement-opsonized particles showed receptor-specific interactions with dendritic cells and B-1 cells [55]. Dextran-coated PLGA carrying SR717 generated a complement component 3 (C3)-enriched corona and enhanced dendritic-cell uptake and immune activation [5]. Thus, opsonization may hinder circulation in some settings while supporting antigen-presenting-cell engagement in others [2,62].

Immunoglobulin effects were similarly selective. High-molecular-weight immunoglobulins contributed to reduced PLGA vascular adhesion under human blood flow [50], and depletion/reconstitution experiments implicated immunoglobulin A (IgA) and immunoglobulin M (IgM), rather than IgG [22]. Anti-PEG IgM could also bind PEGylated nanoparticles and alter the surrounding corona composition [56]. Immunoglobulin adsorption should therefore not be treated as a single mechanistic category. In a PLGA-functionalized multi-walled carbon-nanotube system, plasma proteomics further showed that PLGA functionalization or high PEGylation could favor immunoglobulin adsorption, whereas other formulations favored dysopsonins [63]. Because the carrier core was a carbon nanotube rather than a conventional PLGA nanoparticle, this study is treated as PLGA-containing hybrid evidence rather than as a general PLGA nanoparticle mechanism.

### 3.3. Protein Quantity and Selective Composition

Total adsorbed protein is useful as a formulation-level measurement, but it does not identify which proteins are functionally exposed. Direct PLGA studies show that PEG-associated coatings, PLL-g-PEG, Pluronic F68, and other surface modifications can reduce total adsorption [1,30,42,56,64], and poly(N-isopropylacrylamide) (PNIPAM) versus chitosan conjugation can shift the total protein layer in opposite directions [65]. At the same time, stabilizer changes can alter total bound protein more than overall composition [16], whereas similar gel-level adsorption can conceal species-dependent enrichment and depletion detected by LC-MS/MS [11]. FBS-exposed formulations can differ in both quantity and identity [66].

The key interpretive point is that adsorption mass, protein-class composition, accessibility, and exchange are distinct variables. Chitosan-related PLGA surfaces showed different serum-protein molecular-weight distributions [37], phospholipid and PLGA-PEG matrices produced different hard-corona content [18], and separate PLGA series linked positive charge to high total adsorption [34] but low immunoglobulin adsorption [62]. These findings support selective composition as a distinct variable without implying that any single protein class is universally favorable.

### 3.4. Albumin and Apolipoprotein Recruitment

Albumin appeared repeatedly as both a spontaneously acquired and a deliberately engineered interfacial component. Preformed albumin coronas were generated on PLGA [47], albumin-dominated coronas were observed on some low-adsorption biodegradable polymer particles [67], and PEGylated systems in amniotic fluid could contain high albumin levels [59]. Albumin chemistry also altered adsorption. In comparator-only experiments using dihydrolipoic-acid-coated quantum dots (DHLA-QDs; radius ~5 nm), fluorescence correlation spectroscopy was used to measure the binding of native, succinylated (HSAsuc), and aminated HSA (HSAam) in PBS. The reported binding constants were 6.4 ± 3.6 µM, 19 ± 8 µM, and 22 ± 3 nM, respectively. The authors described HSAsuc as having ~3-fold lower affinity and HSAam as having ~1000-fold higher affinity than native HSA. The measurements used individual HSA variants rather than competitive adsorption from a protein mixture; reversibility experiments were initiated at 90 µM HSA or HSAsuc and 0.9 µM HSAam followed by dilution. Protein modification also altered adsorbed-layer thickness, and binding was reversible. These findings illustrate protein-chemistry-dependent adsorption in this comparator system and do not establish a PLGA-specific affinity relationship [68]. The corresponding adsorbed layers differed in thickness, binding remained fully reversible for all three albumin forms, and cellular uptake varied with the adsorbed albumin form. These findings show that protein chemistry can alter affinity, adsorbed-layer structure, and cellular interaction in that comparator system, but they do not establish a PLGA-specific affinity relationship. Direct PLGA evidence adds a different caution: PLGA nanoparticles can show low albumin adsorption while still inducing measurable changes in albumin structure, and PEGylation can reduce both corona formation and structural perturbation [69].

Apolipoproteins were likewise recurrent and, in some systems, selectively recruited. Spray-dried PLGA and poly(L-lactide) particles showed greater representation of several apolipoproteins than corresponding emulsion-prepared particles [67]. An apolipoprotein E-enriched corona was associated with PLGA-based nanoparticles in another system [19], and a short amyloid-β-derived surface peptide was used to recruit apolipoprotein A-I, apolipoprotein E, and apolipoprotein J while preserving receptor-binding domains; this recruitment principle was explicitly demonstrated on modified PLGA nanoparticles [21]. Together, these studies shift the focus from adsorption as fouling alone to whether recruited proteins retain receptor-accessible function [21].

### 3.5. Hard–Soft Organization, Exchange, Flow, and Analytical Determinants

Corona organization is dynamic. Adsorbed trastuzumab remained associated with PLGA in competing human plasma and serum and behaved as a hard-corona protein, whereas albumin in the comparator condition behaved as a soft-corona protein [43]. Radiolabeled-corona experiments likewise showed greater exchangeability of soft- than hard-corona proteins. In vivo positron emission tomography further showed that soft-corona protein distributed more like free albumin, whereas hard-corona protein followed a nanoparticle-like distribution in lung, liver, and spleen [48]. Matrix composition produced additional hard–soft differences among PLGA, PLGA/cholesterol, and cholesterol particles.

Experimental protocol also shapes the measured protein corona. Venous circulation-mimicking flow preserved material-specific corona signatures while altering subsequent cellular uptake [14], whereas anticoagulant choice influenced the apparent effect of blood exposure on vascular adhesion [15]. Analytical handling introduced additional variability: during human-plasma corona analysis, the use of water versus phosphate-buffered saline for particle dispersion and washing, together with the choice of digestion workflow, altered both the proteins identified and their label-free quantitative intensities [70]. Because these intensities are workflow-dependent relative signals rather than directly comparable absolute quantities, cross-study comparisons require common analytical conditions or appropriate qualification. Thus, the measured corona reflects not only the biological environment but also sample processing and analytical methodology, making cross-study ranking particularly vulnerable to procedural heterogeneity. Table 2 summarizes the robustness tests that most directly challenge conclusions derived from simplified corona models.

## 4. Biointerface-Induced Physicochemical and Functional Reprogramming

Corona acquisition can modify a carrier before any cellular response is measured. Serum proteins increased the hydrodynamic diameter of rosmarinic-acid-loaded PLGA and shifted its surface charge slightly more negative [24]. Plasma proteins produced time-dependent size changes across negatively charged, near-neutral, and positively charged PLGA, with the greatest changes in the positively charged formulation [34]. Concentration-dependent lactoferrin adsorption altered size and zeta potential in a manner consistent with multilayer formation [64]. The particle state presented to a cell is therefore not necessarily the pristine dimension or charge measured immediately after fabrication.

The timing of this remodeling also matters. Poly(zwitterion)-mannose and PEGylated PLGA showed comparable multicomponent aggregation in plasma, yet a short-time difference in corona-associated size was no longer evident at later measurements [44]. PEGylated nanoemulsion-derived PLGA showed no significant size change after an early serum incubation, whereas a polydispersity index of 0.60 ± 0.01 was reported after 5 h [27]. These findings argue against defining biological stability from a single incubation interval.

Corona formation can also affect stability and the preservation of engineered function. Preformed albumin-coated conventional PLGA was more stable than uncoated PLGA [47], PEGylated particles were more stable in amniotic fluid than non-PEGylated comparators [59], and PEGylated docetaxel PLGA/TPGS showed improved stability during serum incubation [4]. Competition from biological components, however, did not invariably erase engineered surface function: CTB retained activity in fresh mouse plasma [41], and adsorbed trastuzumab remained particle-associated and receptor-selective in competing human plasma/serum [43].

Local environmental cues add another layer of reprogramming. The acidity-triggered rational membrane peptide (ATRAM)-BSA-PLGA system underwent intracellular degradation and cargo release after acidity-dependent interaction [71]. Histidine-conjugated star-shaped PLGA/TPGS co-loaded with docetaxel and disulfiram increased release at pH 6.8 [72], whereas RGD-targeted PLGA capsules used radiofrequency-triggered phase transformation of DL-menthol to promote camptothecin release [45]. In tumor extracellular matrix, serum exposure increased PLGA binding to matrix proteins and markedly reduced transport through ex vivo tumor tissue [20]. Biological exposure and local environmental cues can therefore alter cellular recognition, tissue interaction, and activation of engineered release mechanisms.

Evidence from PLGA scaffolds reinforces the principle that adsorbed proteins can mediate downstream cell behavior, while also defining the limits of transferability to nanocarriers. On porous PLGA scaffolds, fetal-calf-serum adsorption increased smooth-muscle-cell attachment relative to fibronectin treatment [73]; a separate PLGA scaffold study identified vitronectin as a predominant adsorbed matrix protein and linked adhesion to defined integrin receptors [74]. In mineral-containing PLGA scaffolds, hydroxyapatite size and crystallinity altered adsorption of adhesive serum proteins and tumor-cell responses [75]. Because scaffold curvature, porosity, and transport differ fundamentally from those of nanoparticles, these studies provide contextual PLGA interface evidence rather than nanoparticle-corona mechanisms.

## 5. Cellular and Immunological Consequences of Acquired Biological Identity

**Figure 1:**
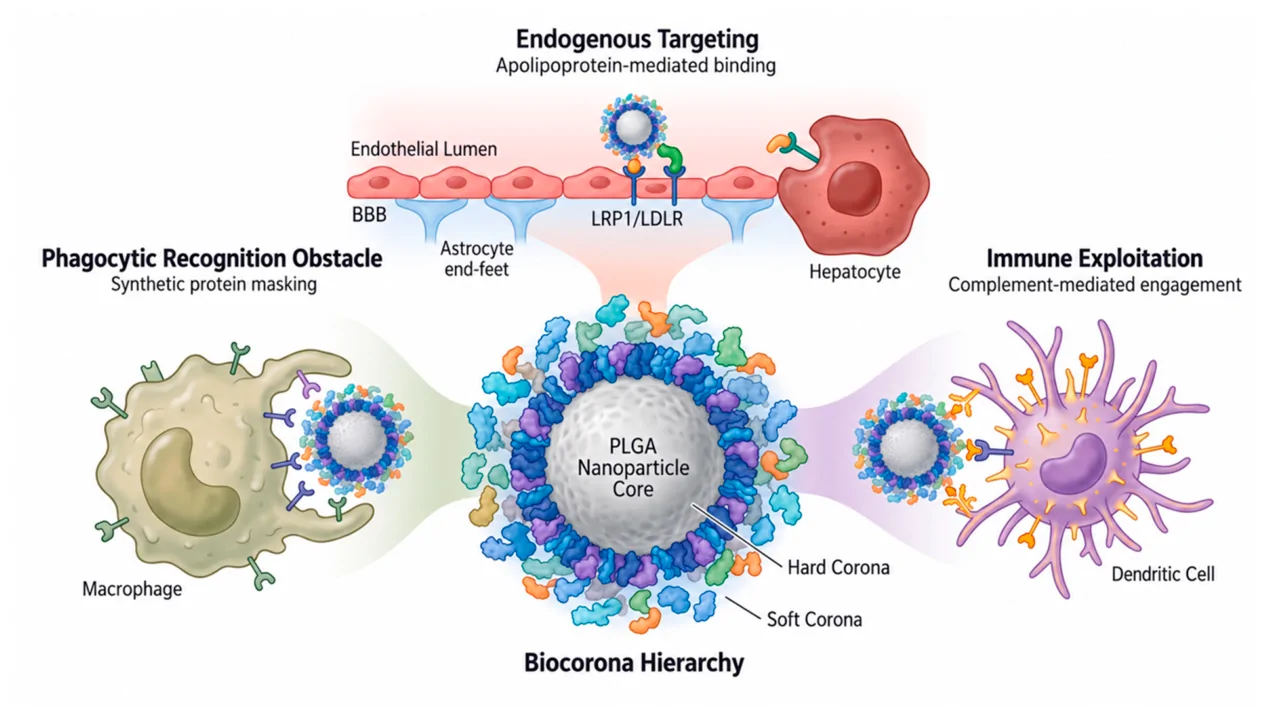
The context-dependent functional valence of the acquired protein corona on PLGA nanocarriers. A corona-bearing carrier can follow divergent trajectories according to protein composition, accessibility, and therapeutic objective: opsonin-rich or altered protein interfaces can promote rapid phagocytic recognition [58,62], whereas immunoglobulin-associated coronas can mask vascular targeting [9,19]; a C3-enriched PLGA interface accompanied enhanced antigen-presenting-cell engagement [5], with complement-receptor dependence supported by a mechanistic comparator [55]; and selectively recruited apolipoproteins can support receptor-mediated endogenous targeting [21]. The figure is conceptual and does not imply that these fates occur simultaneously or with equal probability. Artwork abbreviations: antigen-presenting cell (APC); complement component 3b (C3b); fragment crystallizable (Fc). (Prepared with the assistance of the OpenAI ChatGPT Plus, Gemini Pro, and Gemini Notebook Pro. The authors have reviewed and edited the output and take full responsibility for the content.)

Cellular consequences reflect the combined effects of surface chemistry, acquired proteins, protein accessibility, biological fluid, and receptor repertoire. Corona formation can suppress nonspecific uptake, promote clearance, block an intended receptor interaction, activate antigen-presenting cells, or create a new endogenous targeting route. Figure 1 summarizes this context-dependent functional valence.

### 5.1. Phagocytes, Complement, and Innate Immunity

Phagocyte interactions are particularly sensitive to the acquired interface. Poly(zwitterion)-mannose brush-functionalized PLGA showed substantially greater RAW 264.7 macrophage uptake than mannose-monolayer particles [44]. Conversely, PLL-g-PEG coatings markedly reduced macrophage and dendritic-cell adhesion and phagocytosis, while RGD restored ligand-specific interaction [57]. PEG architecture likewise reduced polymorphonuclear-cell uptake [29], and PEGylation reduced the serum-dependent component of macrophage uptake [76]. Denatured surface-associated albumin promoted mononuclear phagocytic uptake through direct and indirect interactions with scavenger receptor A [46].

Corona composition was also functionally linked to phagocytosis. Nanoparticles carrying an opsonin-rich hard corona were rapidly recognized and internalized by THP-1 monocytes and macrophage-like cells [61], whereas a higher dysopsonin-to-opsonin ratio was inversely related to uptake in another PLGA series [62]. Human serum and FBS generated different leukocyte endocytosis patterns, with complement contributing to uptake under human-serum conditions [13]. PEGylation reduced the serum-dependent component of murine macrophage uptake by suppressing IgG adsorption and complement activation, with complement exerting the stronger influence [76].

Immune recognition, however, is not uniformly undesirable and may be beneficial when aligned with the intended therapeutic function. In a mechanistic complement-opsonized nanocarrier comparator, dendritic-cell uptake required complement receptor 3, whereas B-1-cell binding depended on complement receptors 1 and 2 [55]. In a PLGA immunotherapy system, Dex-PLGA@SR717 formed a C3-enriched corona and was associated with enhanced dendritic-cell uptake and maturation, increased antigen presentation, and T-cell activation against B16F10 melanoma cells in vitro [5]. C3 enrichment should therefore be viewed as context-dependent rather than intrinsically favorable: it accompanied desirable antigen-presenting-cell engagement in this setting, whereas complement also contributed to leukocyte or macrophage uptake in other PLGA systems [10,18]. Because these latter observations do not directly establish systemic clearance, and the SR717 study did not isolate C3 enrichment as the sole causal determinant of immune activation, the C3–immune-response relationship is interpreted as associative [5]. Immune engagement can also be modulated toward distinct functional outcomes; lactoferrin-coated PLGA enhanced phagocytosis of *Escherichia coli* bioparticles while reducing pro-inflammatory cytokine responses in lipopolysaccharide-challenged macrophages and lowering plasma tumor necrosis factor-α in an endotoxemia model [64]. Thus, the design objective is not universally to minimize immune recognition, but to shape it in accordance with the intended biological function.

Additional direct or PLGA-containing evidence shows that innate-cell responses depend on material details beyond a simple corona/no-corona distinction. PLA/PLGA microspheres with different hydrophobicity and end-group chemistry produced different phagocytosis and oxidative-burst responses in murine macrophages and human monocytes [77]. PLGA-coated metal-oxide nanoparticles with different PEG content also produced different neutrophil extracellular-trap responses [78]. Although these systems differ from conventional drug-loaded PLGA nanoparticles, they reinforce the need to measure the immune endpoint relevant to the intended exposure.

Receptor-directed particle surfaces also modulated dendritic-cell phenotype without relying on spontaneous corona engineering. Antibody targeting cluster of differentiation 40 (CD40) and human IgG immobilized on microparticles increased cluster of differentiation 83 (CD83) and cluster of differentiation 86 (CD86) expression and elicited detectable IL-12 secretion. Anti-αvβ3 and anti-αvβ5 promoted maturation with different activation profiles, and covalent antibody attachment generally produced stronger stimulation than physical adsorption [79]. These findings complement the corona-engineering studies by showing that a biological interface can be designed either to limit immune engagement or to present receptor-specific activation cues.

### 5.2. Blood Cells and Vascular Targeting

Interactions with blood cells were strongly surface-dependent. Zwitterionic ionic-liquid-coated PLGA-PEG displayed selective erythrocyte affinity [11], Glyco-IL-modified PLGA showed erythrocyte affinity associated with membrane glucose transporters and the serum-acquired interface [49], and choline-carboxylate coatings enabled blood-cell-specific hitchhiking [80]. Glucose-functionalized PLGA reduced plasma-protein adsorption, complement activation, and platelet plugging while increasing cellular uptake [39]. In contrast, chitosan-related surface chemistries produced different hemocompatibility profiles [37], and surface charge altered hemolysis and coagulation behavior despite no significant cytotoxicity difference among charge groups [34].

These blood-contact studies illustrate why cytotoxicity alone is an inadequate safety surrogate. A formulation can appear noncytotoxic in conventional cell assays yet differ in erythrocyte compatibility, complement activation, platelet plugging, or coagulation time [41,46]. Conversely, deliberate erythrocyte association can serve as a systemic distribution strategy rather than being interpreted automatically as nonspecific adsorption [7,40]. The biological significance of blood-cell binding therefore depends on whether the interaction is damaging, passive, or intentionally exploited for hitchhiking.

Studies of vascular targeting under human flow provide mechanistically supported PLGA evidence. Whole blood markedly reduced endothelial adhesion of vascular-targeted PLGA in a donor-dependent manner [50]. Immunoglobulin depletion restored adhesion, and reconstitution implicated IgA and IgM rather than IgG as major mediators [22]. Anticoagulant choice further altered the apparent phenotype [15]. Together, these findings show that chemical display of a targeting ligand does not guarantee functional accessibility after a human blood-derived corona forms.

### 5.3. Neural, Barrier, and Fluid-Specific Cellular Interactions

PLGA and PLGA-PEG formed distinct serum coronas, and corona-coated PLGA showed cell-type-specific interactions with microglia, neurons, and astrocytes together with inflammatory and viability effects [52]. Corona formation enhanced microglial uptake of fluorinated PLGA and improved fluorine-19 magnetic resonance imaging cell-labeling sensitivity [81]. Cerebrospinal-fluid-derived coronas differed from serum coronas and enhanced brain-cell association under the reported conditions [58], reinforcing the need for anatomically relevant fluids in neuro-nanomedicine.

These findings also caution against treating neuronal uptake as uniformly beneficial. In one study, serum-corona-coated PLGA was readily internalized by microglial and neuronal cells but not by astrocytes, and uptake was accompanied by inflammatory signaling and changes in neuronal viability [52]. In a separate imaging application, enhanced microglial uptake was advantageous because it improved fluorine-19 labeling sensitivity [81]. Increased cellular association may therefore accompany adverse cellular effects, useful imaging contrast, or intended delivery, depending on cell type and application.

Barrier models showed further formulation dependence. CTB-functionalized PLGA retained activity after plasma exposure, crossed an in vitro blood–brain barrier (BBB) model, and targeted glioma and endothelial cells [41]. PEGylated PLGA entered SH-SY5Y cells and promoted crossing/permeabilization in a BBB-on-chip model [27]. In a stringent human iPSC-derived brain microvascular endothelial model, particle association did not by itself predict effects on barrier-forming cells [26]. Elvitegravir-loaded poloxamer-PLGA improved passage across an in vitro BBB model and enhanced subsequent human immunodeficiency virus type 1 suppression in macrophages without compromising barrier integrity [82].

### 5.4. Tumor-Cell Recognition and Intracellular Trafficking

Tumor-cell studies illustrate both masking and recovery of intended interactions. Dextran concentration altered cell-specific uptake of doxorubicin-loaded PLGA across myocardial, breast-cancer, and macrophage models; oxidative membrane damage did not simply parallel uptake or metabolic activity, and deoxyribonucleic acid (DNA) damage was also identified as a relevant toxicity endpoint [83]. TAT-bearing PLGA was designed to avoid interaction at pH 7.4 but recover LS174T interaction at pH 6.5 [17], whereas ATRAM-BSA-PLGA combined macrophage evasion with acidity-triggered membrane interaction [71]. Histidine-conjugated star-shaped PLGA/TPGS improved spheroid penetration and activity in docetaxel-resistant breast-cancer models under an acidic-release design [72].

Protein-bearing and biomimetic interfaces could also preserve or redirect targeting. Albumin-coated PLGA increased internalization and spheroid penetration relative to uncoated particles, whereas albumin-selective particles achieved comparable in vitro performance without a preformed albumin corona [47]. Trastuzumab persisted as a hard-corona-associated targeting protein and supported HER2-selective interaction [43]. Chitosan-coated PLGA-I2BODIPY enhanced photocytotoxicity and reduced macrophage uptake [38]. Anti-HER2 CAR-T-membrane-coated PLGA increased uptake by A549 cells [6], and folate-decorated PEG increased cellular uptake relative to non-targeted PEG in a layer-by-layer system [7].

The tumor microenvironment also presents noncellular barriers that uptake assays cannot capture. Serum exposure increased PLGA binding to tumor extracellular-matrix proteins and markedly reduced transport through ex vivo tumor tissue [20]. This finding provides a mechanistic counterpoint to receptor-targeting strategies: even when receptor binding is preserved in a cell-based assay under otherwise favorable conditions, tissue access may remain limited if the acquired interface increases matrix retention. Tissue penetration therefore represents a distinct level of biological identity between circulation and cellular internalization.

### 5.5. Cellular Function Extends Beyond Uptake

Cellular uptake captures only one dimension of biological identity. Positively charged PLG nanoparticles sustained gene expression in A549 cells longer than polyethyleneimine while showing lower cytotoxicity [1]. LL37-loaded PLGA enhanced wound-related cellular responses [23], and rosmarinic-acid-loaded PLGA was efficiently internalized by SH-SY5Y cells without altering proliferation [24]. PLGA exposure in primary monocytes and MM6 cells did not produce detectable necrosis but altered interleukin-6 secretion and potassium-channel currents [84].

Genotoxicity and oxidative stress likewise did not necessarily coincide. Magnetite-containing nanoparticle systems induced DNA breakage in A549 cells despite low intracellular reactive oxygen species and no significant increase in oxidative DNA damage [85]. These observations reinforce that uptake, viability, inflammatory signaling, oxidative stress, gene expression, barrier integrity, and receptor-specific function are distinct outcomes and should be selected according to the intended application.

## 6. In Vivo Fate, Therapeutic Performance, and Safety

### 6.1. Circulation and Biodistribution

Surface engineering can redirect systemic behavior in vivo, although the effects are not uniform. Glyco-IL-modified PLGA remained longer in the bloodstream and showed reduced liver accumulation relative to control nanoparticles in healthy mice [49]. Albumin coating prolonged circulation of conventional PLGA, whereas albumin-selective chemistry produced comparable in vivo behavior without a preformed albumin corona [47]. PEG-stabilized 2-methoxyestradiol PLGA increased drug half-life/plasma concentration and tumor accumulation relative to casein-stabilized particles despite similar in vitro physicochemical and anti-angiogenic behavior [86]. Choline-carboxylate-coated PLGA underwent blood-cell-specific hitchhiking, reduced residence in plasma, and prolonged overall circulation with altered organ targeting [80].

Corona stability was also associated with protein disposition. In radiolabeled preformed-corona experiments, soft-corona protein behaved more like free albumin, whereas hard-corona protein followed a nanoparticle-like distribution in lung, liver, and spleen [48]. After intra-amniotic delivery, only PEGylated nanoparticles successfully reached fetal organs; PLGA-PEG and poly(amine-co-ester)-PEG systems showed high fetal-lung distribution, whereas PLA-PEG localized to fetal bowel [59].

### 6.2. Tumor Localization and Imaging

Surface modification did not consistently improve tumor delivery. In LS174T tumor-bearing mice, a pH-responsive TAT-bearing formulation reduced pulmonary sequestration but did not meaningfully improve tumor accumulation [17]. In a study of a near-infrared π-conjugated polymer, PLGA-PEG formulations showed favorable phantom performance, yet biodistribution differed and the phospholipid comparator generated a higher tumor signal in vivo [18].

Other formulations showed greater in vivo selectivity. Low-molecular-weight chitosan-coated PLGA-I2BODIPY reduced off-target accumulation and improved tumor selectivity [38]. Anti-HER2 CAR-T-membrane-coated cisplatin PLGA preferentially localized within tumor regions [6], and RGD-targeted camptothecin/DL-menthol PLGA capsules combined with radiofrequency exposure markedly increased tumor retention [45]. PLGA-PEG micelles containing IR-1061 and terminal gadobutrol supported whole-animal near-infrared and magnetic resonance imaging [2].

### 6.3. Therapeutic Performance and Safety

Several engineered PLGA systems produced favorable outcomes in animal models. PEGylated brucine-loaded PLGA reduced tumor growth and mortality relative to free brucine and non-PEGylated nanoparticles [53]. CTB-functionalized paclitaxel-loaded PLGA improved survival in intracranial glioma despite no measurable pharmacokinetic advantage from CTB modification [41]. IL-12-loaded PLGA yielded a reported no-evidence-of-disease outcome in a small treated cohort compared with a historical untreated group, which warrants cautious interpretation [54]. Dextran-coated PLGA@SR717 accumulated in lymphoid organs and suppressed tumor growth after intravenous treatment [5].

Microenvironment-responsive and combination strategies showed additional benefits. pH-responsive docetaxel/disulfiram PLGA increased plasma area under the concentration–time curve (AUC) and tumor delivery and was associated with improved antitumor efficacy, increased intratumoral reactive oxygen species and apoptosis, reduced P-glycoprotein, and prevention of lung metastasis [72]. Radiofrequency treatment of RGD-targeted camptothecin/DL-menthol capsules was accompanied by enhanced retention and markedly improved tumor-growth inhibition [45]. CAR-T-membrane-coated cisplatin PLGA combined preferential localization with reduced tumor growth [6].

Other PLGA studies broadened the range of outcomes without establishing a corona mechanism. PLGA nanocapsules containing the organoselenium analog Di3a showed pH-dependent hemolysis and synergistic activity with doxorubicin or docetaxel in resistant tumor cells [87]. PLGA nanoencapsulation of a thiourea antileishmanial candidate improved selectivity for intracellular amastigotes while reducing macrophage toxicity [88]. In a paclitaxel comparison, PLGA nanoparticles were less cytotoxic to C6 cells at the reported concentration than PLA-Tween 80 formulations [89]. These studies inform translational endpoint selection but are not interpreted as evidence that a specific corona caused the observed therapeutic differences.

Preclinical studies beyond oncology further showed that PLGA biointerface engineering can influence infection, immune modulation, and genetic manipulation. Verteporfin-loaded lecithin-coated PLGA combined with localized ultrasound reduced *Helicobacter pylori* infection in female mice to a degree reported as comparable with standard triple therapy while causing less disruption of the gut microbiota [3]. Lactoferrin-coated PLGA reduced plasma tumor necrosis factor-α in lipopolysaccharide-induced endotoxemia [64]. Antibody-targeted tamoxifen-loaded PLGA also achieved tissue-specific Cre-mediated recombination in vivo [36]. These findings extend the functional consequences of acquired and engineered interfaces beyond tumor accumulation while remaining preclinical rather than clinical demonstrations.

Biodegradability should not be equated with systemic safety. In an anticancer evaluation, ATRAM-BSA-PLGA nanoparticles showed no reported toxicity to healthy tissues [71]. By contrast, in ApoE−/− mice with experimentally induced carotid stenosis, PLGA nanoparticles were administered intravenously at 10 mg/kg every 2 days for 2 weeks and were associated with increased carotid narrowing, intimal thickening, inflammatory responses, smooth-muscle-cell proliferation, abnormal collagen distribution, and endothelial dysfunction. The nanoparticles were prepared from 50:50 PLGA (MW 90,000) using DMSO followed by dialysis to remove the solvent; however, residual DMSO was not quantitatively measured, and PBS served as the control. Thus, the study demonstrates an adverse treatment-associated effect under this dosing and disease-model context but does not isolate intrinsic PLGA chemistry from particle, preparation, repeated-exposure, or model-dependent contributions. These translational findings are summarized in Table 3.

### 6.4. Dose, Schedule, and Exposure Reporting

Dose, administration schedule, and systemic or tissue exposure are integral to the interpretation of in vivo PLGA performance. However, these variables were reported inconsistently across the studies reviewed. In particular, numerical dose was often absent, dosing schedules were incompletely described, and several studies reported biological outcomes without providing a corresponding quantitative measure of nanoparticle or payload exposure. These limitations restrict direct comparison across formulations.

Explicit numerical in vivo dosing was available in only a small subset of the representative studies summarized here. In the metastatic osteosarcoma study, IL-12-containing PLGA nanospheres were administered at 0.1, 1, and 10 mg; at the 0.1 mg level, 75% of treated mice (3/4) showed no evidence of disease, compared with 2/34 animals in a historical untreated control group [54]. In the tumor-immunotherapy study of Dex-PLGA@SR717, the formulation was administered intravenously at an SR717 payload dose of 10 mg/kg and preferentially accumulated in immune-associated organs, particularly the spleen and lymph nodes [5]. Importantly, these values describe different dose bases—an administered nanosphere amount in [54] and an encapsulated-payload dose in [5]—and therefore should not be compared as equivalent nanoparticle doses.

Administration route was reported more frequently than numerical dose, although dosing interval and number of administrations often remained unspecified. Intravenous administration was explicitly reported for ionic-liquid-coated PLGA nanoparticles [80], the vascular-stenosis study, radiolabeled corona-bearing PLGA nanoparticles [48], and Dex-PLGA@SR717 [5]. In the vascular-stenosis study, PLGA nanoparticles were administered by tail vein at 10 mg/kg every 2 days for 2 weeks, with PBS-treated animals serving as controls. The nanoparticles were prepared from 50:50 PLGA (MW 90,000) using DMSO followed by dialysis to remove the solvent; however, residual DMSO was not quantitatively measured. Accordingly, the dose and dosing interval are defined, whereas residual-solvent exposure cannot be quantified from the reported data. The fetal-distribution study was conducted in the context of intra-amniotic nanoparticle delivery and showed that only PEGylated nanoparticles distributed successfully to fetal organs [59]. By contrast, the imaging study in [18] describes the formulations as suitable for parenteral administration but does not specify the actual administration route in the supplied summary. Accordingly, a specific route should not be assigned to that study on the basis of the available information.

Externally applied treatment exposures should also be distinguished from nanoparticle administration routes. In the *H. pylori* study, verteporfin-loaded lecithin-coated PLGA nanoparticles were combined with localized ultrasound at an exposure described as permissible for medical ultrasound devices, but no numerical ultrasound parameter was provided in the supplied summary [3]. Similarly, the RGD-targeted PLGA capsule study employed radiofrequency irradiation to enhance tumor permeability and trigger the DL-menthol phase transition, but the available summary does not provide numerical radiofrequency exposure parameters [45]. Ultrasound and radiofrequency should therefore be described as external treatment exposures rather than administration routes.

Direct pharmacokinetic and biodistribution measurements were available in several studies, although many were reported only comparatively rather than numerically. Glyco-IL-modified PLGA nanoparticles remained in the bloodstream longer and showed significantly reduced liver accumulation relative to control nanoparticles, but no numerical circulation-time or liver-accumulation values were reported [49]. Ionic-liquid-coated PLGA nanoparticles showed reduced residence in plasma together with prolonged overall circulation and formulation-dependent organ-targeting patterns following intravenous administration [80]. Albumin-coated conventional PLGA nanoparticles exhibited longer blood circulation than uncoated nanoparticles, while albumin-selective SA-decorated nanoparticles achieved comparable in vivo performance without requiring a preformed albumin corona [47]. Recruitment of an endogenous apoE-enriched corona likewise prolonged nanoparticle circulation and facilitated tumor accumulation [19].

Drug pharmacokinetics provided more direct measures of systemic exposure in some formulations. PEG-stabilized 2-methoxyestradiol-loaded PLGA nanoparticles showed reduced protein binding and increased drug half-life and plasma concentration relative to the casein-stabilized formulation, resulting in greater tumor accumulation [86]. Similarly, the pH-responsive docetaxel/disulfiram formulation produced significantly higher plasma area under the concentration–time curve and greater tumor drug delivery than comparator conditions in an orthotopic breast-cancer model [72]. The available summaries, however, do not provide the corresponding numerical pharmacokinetic values for either study. In the CTB-functionalized PLGA-paclitaxel study, pharmacokinetic profiles were assessed and CTB modification was reported not to alter those profiles relative to unmodified PLGA nanoparticles, but numerical pharmacokinetic parameters were likewise not provided in the supplied summary [41].

Imaging and organ-distribution studies supplied additional measures of in vivo exposure. Positron-emission-tomography analysis of intravenously administered PLGA nanoparticles carrying radiolabeled protein coronas showed that soft-corona proteins predominantly exhibited activity in the blood and kidneys, whereas hard-corona proteins followed nanoparticle-like biodistribution with accumulation in the lungs, liver, and spleen [48]. In 4T1 tumor-bearing mice, chitosan-coated PLGA nanoparticles reduced accumulation relative to uncoated nanoparticles by 10.2-fold in lymph nodes, 2.1-fold in spleen, 1.3-fold in liver, 22.7-fold in skin, and 4-fold in eyes while improving tumor-targeting selectivity [38]. In the intra-amniotic-delivery study, PLGA-PEG and PACE-PEG nanoparticles showed high distribution to fetal lung, whereas PLA-PEG nanoparticles distributed exclusively to fetal bowel [59]. In the murine HeLa xenograft imaging study, PEG(2 kDa)-PLGA(4 kDa) particles accumulated more rapidly in the liver, whereas PEG(2 kDa)-DPPE particles showed greater tumor localization [18]. CAR-T-cell-membrane-coated PLGA nanoparticles also showed preferential localization within tumor regions in a subcutaneous lung-cancer model [6], while radiofrequency treatment increased the retention of RGD-targeted PLGA capsules in HeLa tumors by more than 8-fold in vitro and in vivo [45]. Live-mouse bimodal imaging with PLGA-PEG micelles additionally permitted visualization of veins and organs, although the supplied summary does not report a numerical dose or quantitative tissue-exposure measurement [2].

Downstream pharmacodynamic, efficacy, and safety endpoints should not be treated as substitutes for exposure measurements. Examples include tumor growth and mortality [53], survival in intracranial glioma [41], no-evidence-of-disease outcomes [54], infection control and microbiota effects [3], tissue-specific Cre recombination [36], antitumor activity and healthy-tissue toxicity [71], plasma TNF-α reduction [64], vascular pathology, tumor suppression [5], tumor-growth reduction [6], and tumor-growth inhibition [45]. These endpoints are important for interpreting biological response, but without accompanying dose and pharmacokinetic or biodistribution data they cannot independently establish the magnitude or duration of nanoparticle exposure.

For translational interpretation, future in vivo studies should therefore distinguish nanoparticle or formulation dose from payload dose, report the dose basis and units, specify the administration route, number of administrations, dosing interval, and total exposure period, and pair these variables with at least one direct exposure measure where feasible. Relevant measures include blood or plasma concentration–time profiles, area under the concentration–time curve, half-life, quantitative organ or tumor burden, and particle- or payload-equivalent tissue exposure. Where only a downstream pharmacodynamic, efficacy, or safety endpoint is available, it should be identified as such rather than classified as an exposure metric. Consistent reporting of these parameters would substantially improve cross-study interpretation of PLGA efficacy, biodistribution, and safety.

## 7. Translational Design Principles and Evidence Gaps

**Figure 2:**
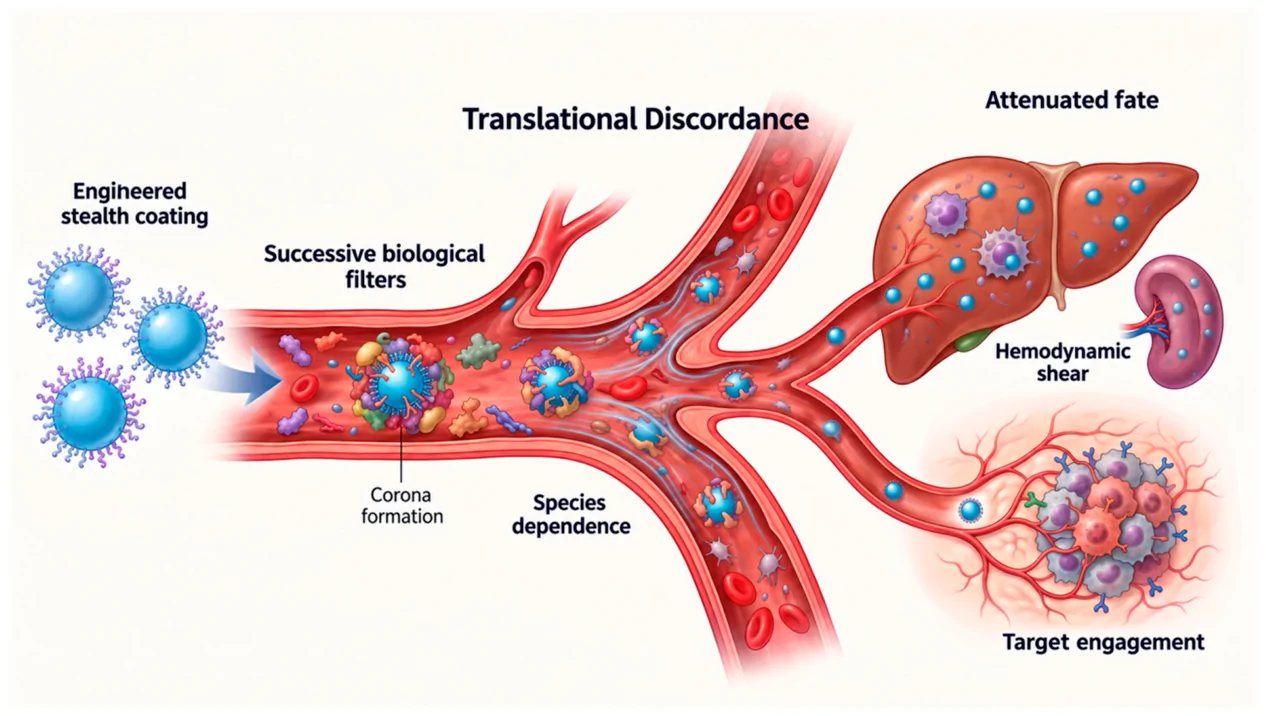
A heuristic biological stress-test map for PLGA function and translational decoupling. Engineered PLGA properties encounter multiple, partly parallel and reversible biological trajectories: biofluid-specific corona formation and exchange; shear- and donor-dependent blood interactions; immune-cell recognition or blood-cell hitchhiking; endothelial and tissue-barrier transport; extracellular-matrix sequestration; and organ-level clearance or retention. Species and donor dependence [9,12,13], dynamic flow [14], anticoagulation [15], tissue sequestration [20], and biodistribution [17,18] can each redirect an upstream design advantage. The diagram is heuristic and does not imply that all particles pass through these states in a fixed order or that a single corona persists unchanged across compartments. (Prepared with the assistance of the OpenAI ChatGPT Plus, Gemini Pro, and Gemini Notebook Pro. The authors have reviewed and edited the output and take full responsibility for the content.)

Overall, the evidence supports a biological stress-test model for PLGA development. The engineered pre-corona state is challenged by biofluid exposure and competitive adsorption, dynamic blood and cellular interactions, tissue barriers and sequestration, and whole-organism distribution and clearance. These processes can occur in parallel, recur as particles enter new compartments, and partially reverse as proteins exchange; Figure 2 should therefore be read as a heuristic map of possible transitions rather than as a deterministic linear sequence.

The evidence hierarchy is essential when translating general nanobio principles into PLGA claims. Direct PLGA findings can establish formulation-specific effects, and depletion/reconstitution or receptor perturbation provides the strongest mechanistic support when available [22]. Associative PLGA proteomics can identify plausible mediators but should not be treated as causal without perturbation. Comparator-only evidence can generate hypotheses but cannot establish a PLGA-specific mechanism. Such studies show, for example, how tunable polymer coatings or PEO architecture can alter adsorption and circulation [37,91], how charge or surface amines can alter plasma corona and organ distribution [91], how particle size can change immune phenotype or the effectiveness of PEG/albumin passivation [35,92], how protein coatings can alter short-term cellular selectivity [93], and how formulation differences can affect uptake and responses in magnetic or cerium-oxide systems [92,94]. Reconfigurable antiopsonic carrier manufacturing [95], corona-linked organ targeting in non-PLGA polymer systems [96,97], and quantitative tumor-targeted micelle studies [98] likewise inform design principles without showing that the same mechanisms operate in PLGA.

In direct PLGA studies, the engineered pre-corona identity and the acquired biological identity should be characterized separately. Initial descriptors such as particle size, polydispersity, zeta potential, PEG architecture, surface coating, ligand status, and payload remain essential, but these properties should be reassessed after exposure to the intended biological environment [6,8,11,15,32,41,99]. This distinction is particularly important because the acquired interface depends strongly on biological context: human and mouse serum produce different corona profiles [10,11], human serum and fetal bovine serum can lead to different leukocyte-uptake patterns [13], cerebrospinal fluid generates a distinct environment for brain-relevant systems [58], and anticoagulant choice can alter apparent vascular-adhesion behavior [15]. Consistent with this context-dependent view, a five-module framework has been proposed that integrates nanoparticle synthesis, exposure to a relevant human biological fluid, target-tissue considerations, corona characterization, and risk assessment [100]. Together, these approaches emphasize that PLGA nanocarriers should be evaluated not only by their manufactured properties, but also by the biological identity they acquire under application-relevant conditions.

Methodological standardization is therefore central to mechanistic inference. Flow versus static exposure changes corona behavior [14], washing and digestion workflows influence protein identification and label-free intensities [70], and donor variability substantially changes vascular-targeting performance [50]. Reports should specify fluid source and concentration, donor/species, incubation time and temperature, flow state, anticoagulant, separation/washing conditions, proteomic workflow, particle and payload dose, dosing schedule, and exposure metric. Total protein adsorption should be interpreted alongside selective composition, exchange, and functional perturbation rather than used as a surrogate for biological quality.

Future PLGA design should move beyond empirical stealth optimization toward predictive corona engineering. Several elements of that strategy are already evident in the reviewed literature: small changes in ionic-liquid chemistry generated distinct serum coronas and were analyzed with machine-learning methods [10]; human donor variability altered vascular targeting [50]; compartment-specific fluids changed acquired identity [8,14,15]; apolipoprotein recruitment supported targeting [21]; and C3 enrichment accompanied enhanced immune engagement [5]. These findings support future models that predict a distribution of biological identities from formulation descriptors, donor- or disease-state biofluids, and dynamic exposure conditions rather than seeking a single universal corona. Machine-learning-guided formulation optimization is therefore a plausible extension of the demonstrated analytical approaches, but prospective artificial-intelligence-designed PLGA formulations are not yet established by the evidence synthesized here.

Patient- and disease-specific design represents another logical translational direction. Donor-dependent blood effects [50], species differences [10,11], and compartment-specific coronas [8,14,15] indicate that a formulation optimized in pooled or xenogeneic serum may not preserve its function in the intended population. Programmable biological identities may be created deliberately through persistent proteins, biomimetic membranes, or ligands that recruit endogenous proteins [3,27,54]. Nucleic-acid aptamers are conceptually compatible as a future ligand class, although this review does not treat them as an established PLGA corona strategy.

The principal translational limitation is not simply heterogeneity among studies but incomplete linkage between exposure, acquired interface, mechanism, and outcome. Across the preclinical studies discussed here, changes in circulation, tumor accumulation, area under the concentration–time curve, organ distribution, or therapeutic activity are not consistently integrated with quantified exposure and mechanistic measurements [3,23,40,59,64,77]. Human blood and human-cell studies strengthen mechanistic relevance but do not establish patient-level efficacy [9,10,19,22]. The field will become more predictive when individual experiments connect defined formulation variables to measured corona state, causal functional perturbation, quantified in vivo exposure, and the intended efficacy or safety endpoint.

## 8. Conclusions

PLGA formulation establishes a starting bias, not a fixed biological identity. Across these studies, the same engineered feature can remain functional, become masked, recruit a new endogenous function, or become irrelevant as the carrier encounters changing proteins, flow, immune cells, tissue matrices, and organ-level clearance. Translational failure arises when an upstream optimization is treated as invariant across those transitions; translation improves when the intended function is tested as a property of the acquired interface under the exposure conditions in which the carrier must operate.

The key conceptual shift is therefore from optimizing a pristine nanoparticle to designing for functional robustness under biological reprogramming. No single coating, corona composition, or degree of protein adsorption is universally optimal. What matters is whether a formulation produces a reproducible range of biological identities that remains compatible with the therapeutic objective, dose, route, patient context, and safety constraints. The evidence remains preclinical and does not support clinical efficacy claims or meta-analytic effect estimates, but it provides a clear basis for more predictive PLGA development, connecting the engineered state, acquired interface, causal mechanism, quantified exposure, and downstream outcome within the same translational framework.

## Figures and Tables

**Table 1:** Quantitative design–response relationships that define boundary conditions for PLGA biological identity. Purpose: Rather than classifying formulations, the table addresses design questions and emphasizes quantitative thresholds or contrasts that inform interpretation of the acquired biological interface.

Design Question	Quantitative Evidence	Consequence for Acquired Identity/Function	Boundary Condition/Interpretation	Refs.
Is increasing PEG content continuously beneficial?	In a PEG–polyester series that included PLGA, PEG molecular weights of 2000–20,000 g/mol and PEG contents of 0.5–20 wt% were examined. For the specified poly(lactic acid) (PLA)-based PEG-PLA45K formulation, maximal protein resistance occurred at 5000 g/mol PEG and 2–5 wt% PEG; increasing PEG above 5 wt% produced no further reduction. This optimum corresponded to approximately 1.5 nm between terminal PEG chains.	The same PEG range minimized both protein adsorption and polymorphonuclear-cell uptake.	PEGylation shows a formulation-dependent saturation threshold; this numerical optimum should not be generalized to every PLGA architecture.	[29]
How large can the effect of surface passivation on protein adsorption be?	PLL-g-PEG reduced adsorption on PLGA microspheres by ~2 orders of magnitude, and 0.5% Pluronic F68 reduced BSA adsorption by ~50%. In the PLGA-PEG brucine series, serum-protein adsorption was 14.9 ± 1.08 to 25.5 ± 1.5 µg/mg, versus 44.7 ± 5.0 to 74.7 ± 3.8 µg/mg for corresponding non-PEGylated PLGA.	Surface passivation can substantially reduce protein adsorption.	Values should not be compared directly across studies. The brucine values are mass-normalized, not surface-area-normalized, so they support a within-study comparison but not a cross-study benchmark.	[42,55,56]
Does particle size independently determine protein adsorption within a conventional PLGA nanoparticle range?	PLGA and PLGA-PEG particles were directly compared at 100 and 200 nm. Protein adsorption was independent of size over this interval, whereas PEGylation significantly reduced total bound protein and depleted immune-response-associated proteins.	Within the tested range, PEGylation rather than particle diameter substantially affected adsorption.	The absence of a size effect over 100–200 nm does not exclude size dependence outside that interval.	[25]
Can manufacturing alter the biological starting state before corona formation?	Microfluidic fabrication of palmitoylated LL37-loaded PLGA produced particles of 102.3 ± 2.0 nm, compared with 189.3 ± 3.4 nm after bulk nanoprecipitation, with improved uniformity and prolonged peptide release.	Fabrication materially changed the pre-corona dimensions and stability from which biological exposure began.	Manufacturing is an upstream determinant of biological identity even when the nominal polymer and payload are unchanged.	[23]
Can receptor-directed uptake increase without a parallel increase in plasma aggregation?	Poly(zwitterion)-mannose brush PLGA produced approximately fourfold greater RAW 264.7 uptake than mannose-monolayer particles. Brush and PEGylated controls each showed approximately 760 aggregated particles per 10^5^ particles. Corona-associated size was assessed at 0, 8, and 15 h; a roughly 130 nm short-time difference was no longer evident later.	Macrophage targeting increased without evidence of greater multicomponent aggregation than in the PEGylated comparator.	Increased cell association cannot automatically be attributed to poorer colloidal stability; receptor presentation and temporal remodeling should be considered separately.	[44]
Can low nonspecific phagocytosis coexist with ligand-specific interaction?	PLL-g-PEG reduced phagocytosis of coated PLGA microspheres to approximately 10% in serum. Addition of RGD restored ligand-specific macrophage and dendritic-cell interaction, whereas the inactive Arg-Asp-Gly (RDG) control did not.	A cell-repellent background suppressed nonspecific phagocytosis while retaining receptor-specific interaction.	Stealth and active targeting are not intrinsically incompatible, but targeting should be tested after serum exposure.	[57]
Can surface chemistry simultaneously change formulation quality and blood compatibility?	In uncoated and carboxymethyl-chitosan-coated PLGA, extraneous paclitaxel precipitates accounted for >25% of the drug; glycol-chitosan reduced this proportion to approximately 5%. Carboxymethyl-chitosan produced >10% hemolysis, whereas glycol-chitosan showed superior hemocompatibility at a comparable concentration.	Surface modification altered both formulation quality and blood compatibility.	Surface engineering should be evaluated as a coupled formulation-biointerface intervention rather than optimized against a single endpoint.	[37]
Can a targeting element remain functional after prolonged competitive protein exposure?	CTB retained biological activity after 24 h in fresh mouse plasma and did not measurably alter corona composition, macrophage phagocytosis, or pharmacokinetics relative to unmodified PLGA. In a separate system, adsorbed trastuzumab behaved as a persistent hard-corona protein and retained HER2-selective interaction.	Competitive biological exposure did not inevitably abolish engineered ligand function.	Ligand persistence is system specific; direct post-corona functional testing is more informative than assuming either masking or preservation.	[47,54]

**Table 2:** Robustness tests that determine whether an experimentally measured PLGA biological identity is physiologically meaningful. Purpose: The table identifies experimental stress tests that can overturn conclusions obtained under simplified conditions, emphasizing human relevance, donor dependence, dynamic exposure, and analytical reproducibility.

Robustness Question	Experimental Stress Test/Quantitative Anchor	Observed Consequence	Implication for Design and Interpretation	Refs.
Does biological identity remain stable over time in plasma?	Poly(zwitterion)-mannose and PEGylated PLGA were evaluated at 0, 8, and 15 h in plasma. Approximately 760 particles per 10^5^ particles were present in multicomponent aggregates for both formulations; a roughly 130 nm short-time size difference was no longer evident later.	Early physicochemical differences were not necessarily retained during longer exposure.	A single early measurement may not represent the interface observed after prolonged plasma exposure.	[44]
Do later serum measurements reveal changes not evident at early time points?	PEGylated PLGA in 10% FBS at 37 °C showed no significant size change after 2 h; after 5 h, the reported diameter was 101.4 ± 15.3 nm with a polydispersity index of 0.60 ± 0.01.	The early 2 h size result did not establish the later reported size distribution.	Stability claims should specify biological-fluid concentration and exposure duration; an early size measurement alone may be insufficient.	[27]
How large can human donor variability be?	Under human blood flow, vascular-targeted PLGA adhesion was reduced by up to 90% relative to buffer. A reduction > 80% occurred in approximately 60% of unique donor samples, whereas other donors produced moderate or negligible inhibition.	The same particle showed markedly different vascular-targeting performance depending on donor blood.	Human donor variability is a biological variable rather than experimental noise; single-donor studies can misestimate targeting robustness.	[50]
Can a specific corona protein class be causally linked to loss of targeting?	Removing immunoglobulins largely restored endothelial adhesion. Reintroducing IgA or IgM produced approximately 40–70% lower adhesion than an immunoglobulin-deficient corona, whereas high-concentration IgG did not significantly suppress adhesion.	The phenotype could be narrowed to specific immunoglobulin classes rather than total immunoglobulin adsorption.	Depletion/reconstitution provides stronger mechanistic evidence than correlation between proteomic abundance and function.	[22]
Does blood-processing chemistry change the apparent phenotype?	PLGA, poly(lactic acid) (PLA), poly(ε-caprolactone), silica, and polystyrene carriers were compared under non-anticoagulated, heparinized, and citrated conditions. Non-anticoagulated blood produced strong adhesion reduction, whereas anticoagulant-containing blood produced only moderate or minimal effects depending on material; PLGA was the most sensitive material tested.	The apparent effect of the corona on vascular targeting changed with anticoagulant choice.	Anticoagulant choice is part of the experimental model and should be reported and justified.	[15]
Does a stringent human barrier model expose effects missed by uptake measurements alone?	Human induced pluripotent stem cell (iPSC)-derived brain microvascular endothelial cells formed barriers with transendothelial electrical resistance reaching 4000 Ω·cm^2^. Comparable amounts of 50 and 100 nm particles interacted with endothelial cells, yet 100 nm PLGA altered barrier-forming cells in a way not observed with the other tested formulations.	Similar particle association did not imply equivalent endothelial-cell responses.	Uptake or association should not be used as a surrogate for equivalent effects on barrier-forming cells.	[26]
Can blood compatibility diverge substantially despite broadly similar nanocarrier intent?	A zwitterionic ionic-liquid PLGA-PEG system produced approximately 5% human hemolysis. In a separate PLGA surface-chemistry comparison, carboxymethyl-chitosan particles produced >10% hemolysis, whereas glycol-chitosan showed superior hemocompatibility.	These examples show that surface chemistries intended to alter biointeractions can differ in measured blood compatibility.	Hemocompatibility requires direct testing; cross-study values should not be treated as a head-to-head comparison.	[13,43]
Can analytical processing alter the corona that is reported?	Human-plasma samples were examined after dispersion/washing in water versus phosphate-buffered saline and with four digestion procedures. Phosphate-buffered saline yielded fewer high-isoelectric-point proteins and fewer immunoglobulins; filter-aided sample preparation produced the greatest number of identified proteins and the highest label-free quantification intensities.	Protein identification and quantitative abundance depended on the sample-processing workflow.	The reported corona profile is method dependent; cross-study comparison requires harmonized or explicitly reported methods.	[70]
Does a static corona necessarily represent a circulating one?	A venous circulation-mimicking system incorporated shear and dynamic protein exchange. PLGA and other soft carriers retained material-specific signatures under flow, and preformed flow coronas markedly reduced A549 uptake.	Dynamic exposure generated material-specific coronas with measurable consequences for cell uptake.	Static incubation should be complemented by dynamic models when circulation is central to the intended use.	[14]
Do anatomically relevant fluids produce interchangeable biological identities?	Serum versus cerebrospinal fluid generated distinct enrichment/depletion patterns; human serum and FBS differed in composition and leukocyte endocytosis; and amniotic- and vitreous-fluid studies showed additional fluid-specific behavior.	Corona composition and subsequent cellular association depended on the biological compartment used to generate the interface.	Fluid selection should follow the route of administration and target tissue; these studies support context-specific interpretation rather than a single quantitative ranking across fluids.	[8,10,14,15,16]

**Table 3:** Quantitative translational concordance and discordance across the PLGA design–biointerface–outcome continuum. Purpose: The table tests whether apparently favorable upstream properties predict downstream outcomes and highlights where biological exposure amplifies, redirects, or decouples engineered function.

Translational Question	Upstream Observation	Quantitative Downstream Benchmark	Concordance and Interpretation	Refs.
Is improved pharmacokinetics required for improved therapeutic efficacy?	CTB retained activity after plasma exposure, crossed an in vitro BBB model, and targeted glioma/endothelial cells, but did not measurably alter corona composition, macrophage phagocytosis, or pharmacokinetics relative to unmodified PLGA.	Survival was 34 days for CTB-functionalized PLGA nanoparticle/paclitaxel versus 29 days for unmodified PLGA nanoparticle/paclitaxel, 24 days for Taxol, and 21 days for saline.	Discordant with a pharmacokinetic-only model: survival improved despite no measured pharmacokinetic difference, so the efficacy difference was not explained by the reported systemic pharmacokinetic measurements alone.	[41]
Does superior intrinsic imaging performance predict superior tumor imaging?	PLGA-PEG formulations produced approximately 30% higher phantom signal-to-background than the PEGylated phospholipid comparator; the PEG(2 kDa)-PLGA(4 kDa) formulation also produced the highest phantom photoacoustic amplitude.	In mice, PEG(2 kDa)-PLGA(4 kDa) accumulated more rapidly in liver, whereas the phospholipid comparator localized more strongly to tumor; the PLGA-PEG formulation generated a lower tumor imaging signal.	Discordant: superior phantom performance did not translate into superior tumor imaging because biodistribution differed among formulations.	[18]
Can an engineered biointerface translate into quantitatively reduced off-target exposure?	Low-molecular-weight-chitosan coating reduced serum-protein adsorption and macrophage uptake while enhancing tumor-cell photocytotoxicity.	Relative to uncoated PLGA-I2BODIPY, accumulation decreased 10.2-fold in lymph nodes, 2.1-fold in spleen, 1.3-fold in liver, 22.7-fold in skin, and 4-fold in eyes; both nanoparticle formulations were more efficacious than free I2BODIPY.	Concordant: reduced nonspecific biointeraction was accompanied by substantial reductions in several off-target compartments and maintained or enhanced anticancer activity.	[38]
Can an external physical intervention overcome biological loss of molecular targeting?	RGD targeting was combined with radiofrequency-enhanced tumor permeability and radiofrequency-triggered DL-menthol phase transition/camptothecin release.	Radiofrequency exposure increased HeLa-tumor retention by >8-fold and produced >10-fold improvement in tumor-growth inhibition.	The combined intervention was associated with substantial gains in retention and tumor-growth inhibition; the observed effect should not be attributed to any single component without component-specific evidence.	[45]
Can an immune-promoting corona be therapeutically useful rather than a liability?	Dextran-coated PLGA@SR717 formed a C3-enriched human-serum corona, increased dendritic-cell uptake, maturation, and antigen presentation, and preferentially accumulated in spleen and lymph nodes.	Intravenous SR717 was administered at 10 mg/kg and significantly suppressed tumor growth.	Directionally concordant: the C3-enriched corona accompanied enhanced immune engagement and tumor suppression, but the association does not establish C3 enrichment alone as the cause of efficacy.	[5]
Can a favorable animal efficacy signal still require cautious interpretation?	IL-12 PLGA optimization increased encapsulation efficiency from 28.3 ± 6.10% in a surfactant-only formulation to 85.4 ± 2.19% when surfactant and FBS were incorporated during formulation; FBS was subsequently replaced with mouse serum albumin for in vivo use.	At 0.1 mg, 75% (3/4) of mice showed no evidence of disease versus 2/34 animals in a historical untreated control group.	Promising but methodologically limited: the comparator was historical and the treated sample was very small, so the result should not be treated as a controlled effect-size estimate.	[54]
Does biomimetic targeting persist from cell interaction to whole-animal localization?	Anti-HER2 CAR-T-membrane-coated cisplatin PLGA increased A549 uptake and inhibited multiple HER2-positive cell types; cisplatin release persisted for 21 days under physiological conditions.	In a subcutaneous lung-cancer model, membrane-coated particles preferentially localized to tumor and significantly reduced tumor growth.	Directionally concordant: related in vitro and in vivo targeting phenotypes were observed.	[6]
Does biodegradability establish systemic safety?	ApoE−/− mice with carotid stenosis received PLGA nanoparticles intravenously at 10 mg/kg every 2 days for 2 weeks; PBS served as control.	Treatment was associated with increased carotid narrowing, intimal thickening, inflammation, and endothelial dysfunction.	PLGA was prepared in DMSO followed by dialysis, but residual DMSO was not quantified. The study therefore supports a model- and exposure-specific safety signal, not attribution to intrinsic PLGA chemistry alone.	[90]

## Data Availability

No new data were created or analyzed in this study. Data sharing is not applicable to this article.

## Glossary and Abbreviations

Dysopsonin	protein or other biomolecule that can reduce phagocytic recognition or uptake of a particle, depending on biological context
Hard corona	relatively tightly bound biomolecular layer with longer residence at the nanoparticle surface and slower exchange with the surrounding medium
Opsonin	biomolecule, such as an immunoglobulin or complement component, that can promote recognition and uptake by phagocytic cells
Soft corona	more weakly associated, dynamically exchanging biomolecular layer surrounding the particle or hard corona
Vroman effect	time-dependent competitive adsorption in which initially adsorbed, often abundant proteins can be displaced by proteins with higher surface affinity
APC	antigen-presenting cell
ApoE−/−	apolipoprotein E-deficient (knockout) genotype
ATRAM	acidity-triggered rational membrane peptide
AUC	area under the concentration–time curve
BBB	blood–brain barrier
BSA	bovine serum albumin
C3	complement component 3
C3b	complement component 3b
CAR-T	chimeric antigen receptor T cell
CD40	cluster of differentiation 40
CD83	cluster of differentiation 83
CD86	cluster of differentiation 86
CTB	cholera toxin subunit B
DMSO	dimethyl sulfoxide
DNA	deoxyribonucleic acid
FBS	fetal bovine serum
Fc	fragment crystallizable
Glyco-IL	sugar-based ionic liquid
HER2	human epidermal growth factor receptor 2
I2BODIPY	diiodinated boron-dipyrromethene
IgA	immunoglobulin A
IgG	immunoglobulin G
IgM	immunoglobulin M
IL-12	interleukin-12
iPSC	induced pluripotent stem cell
LC-MS/MS	liquid chromatography-tandem mass spectrometry
NR	not reported
PBS	phosphate-buffered saline
PEG	poly(ethylene glycol)
PEO	poly(ethylene oxide)
PLA	poly(lactic acid)
PLG	poly(D,L-lactide-co-glycolide)
PLGA	poly(lactic-co-glycolic acid)
PLL-g-PEG	poly(L-lysine)-graft-poly(ethylene glycol)
PNIPAM	poly(N-isopropylacrylamide)
RDG	Arg-Asp-Gly
RGD	Arg-Gly-Asp
TAT	transactivator-of-transcription peptide
TPGS	D-α-tocopheryl polyethylene glycol succinate
